# Intraparenchymal Mucosa-Associated Lymphoid Tissue Lymphoma: A Case Report

**DOI:** 10.7759/cureus.28301

**Published:** 2022-08-23

**Authors:** Pedram Laghaei Farimani, Vishwathsen Karthikeyan, Mostafa Fatehi, Adrian Levine, Graham W Slack, Ian R Mackenzie, Charles Haw

**Affiliations:** 1 Medicine, University of British Columbia, Vancouver, CAN; 2 Neurosurgery, Vancouver General Hospital, Vancouver, CAN; 3 Neurosurgery, University of Toronto, Toronto, CAN; 4 Pathology and Laboratory Medicine, Vancouver General Hospital, Vancouver, CAN; 5 Pathology and Laboratory Medicine, University of British Columbia, Vancouver, CAN

**Keywords:** surgical case report, intraparenchymal, marginal zone b cell lymphoma, cns lymphoma, cns lesions, mucosa-associated lymphoid tissue (malt), mucosa-associated lymphoid tissue (malt) lymphoma

## Abstract

Marginal zone B-cell lymphoma (MZBCL) of mucosa-associated lymphoid tissue (MALT) type, which is primary to the central nervous system (CNS), is a rare lesion, with those originating within the parenchyma even more so. We present the case of a 64-year-old male with weakness in the left hand and focal motor seizures of his arm, who was found to have a right frontal intraparenchymal lesion. Following resection, histopathological and immunohistochemical evaluations were completed, leading to a diagnosis of a primary CNS MZBCL of MALT type in the context of a negative workup of systemic disease. Neuroimaging, histopathological, and immunohistochemical findings, as well as a comprehensive literature review of similar cases, are discussed.

## Introduction

Primary central nervous system lymphoma (PCNSL) is an aggressive yet rare variant of extranodal non-Hodgkin lymphomas, accounting for roughly 4% of primary and malignant tumors of the CNS [1]. They mainly arise from the brain, leptomeninges, spinal cord, and vitreoretinal compartment of the eye [2]. Approximately 90% of cases reported as PCNSL are diffuse large B-cell lymphomas, leaving small B-cell lymphomas in the minority [3].

Under the category of small B-cell lymphomas, marginal zone lymphomas (MZL) represent the majority of neoplasms that are primary to the CNS [4]. Mucosa-associated lymphoid tissue (MALT) lymphoma as a subtype of MZL, originally described as low-grade lymphomas within the gastrointestinal tract, is primarily found within the stomach but is also commonly found within salivary glands, the thyroid, ocular adnexa, lungs, and breasts [5,6]. However, primary CNS MALT lymphomas are rare.

Tu et al. reported 15 primary CNS MZL cases, of which 93% were dural-based lesions mimicking meningiomas, arising from sites including the convexity of the brain, falx, tentorium, middle skull base, ventricles, and spinal dura mater [7]. Primary CNS MZL lesions that arise from the parenchyma, however, are exceptionally rare and can be misdiagnosed as gliomas in certain patients [8].

We present a case of a patient with primary CNS intraparenchymal MALT lymphoma with an immunohistochemical profile. Furthermore, a review of the literature on similar cases, including treatment options and outcomes, is discussed. To our knowledge, the present case represents the first reported instance of a patient with such a lesion to be managed solely with surgical resection.

## Case presentation

**Figure 1:**
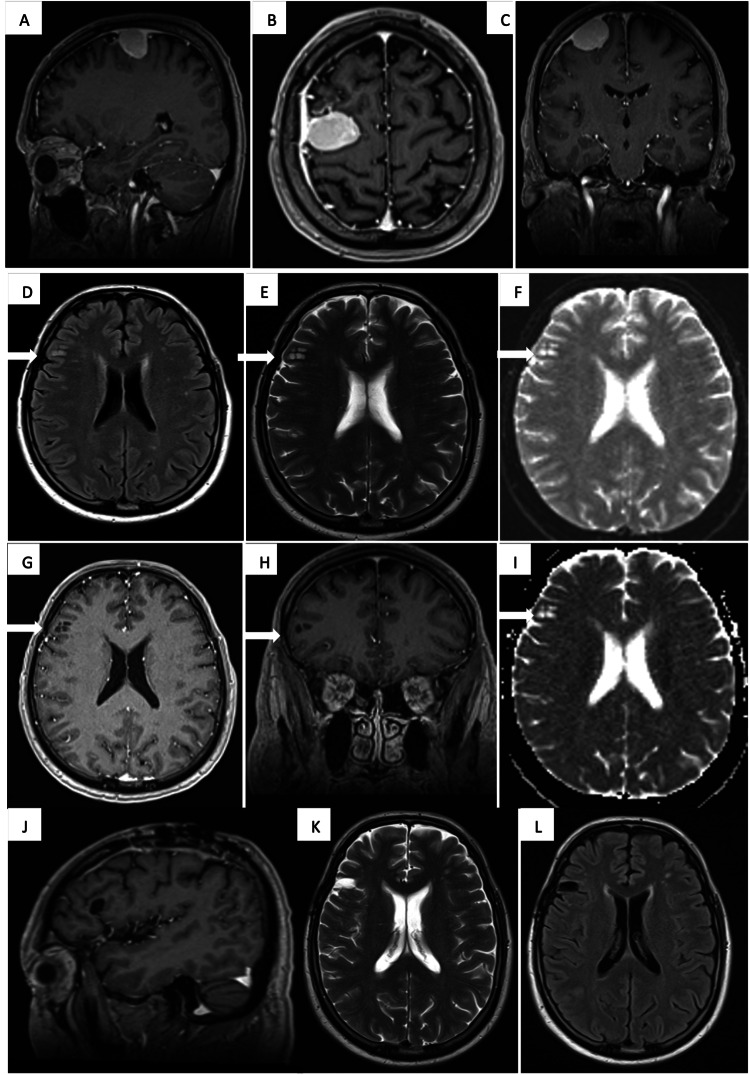
Magnetic resonance imaging Panels A, B, and C demonstrate right frontal meningioma. Panels D and E represent the FLAIR and T2 sequence where the white arrows point to the lesion diagnosed as MZBCL of MALT type while panels F and I are the diffusion-weighted image and apparent diffusion coefficient. Axial and coronal T1 with gadolinium are shown in panels G and H, respectively. Panels J, K, and L show a follow-up MRI obtained nine months after surgery, which confirms there is no residual or recurrent lesion. FLAIR: fluid-attenuated inversion recovery; MZBCL: marginal zone B-cell lymphoma; MALT: mucosa-associated lymphoid tissue

A 64-year male with a history of rheumatoid arthritis and anti-phospholipid syndrome presented with mild left-hand weakness and a focal motor seizure involving his arm. The patient was receiving hydroxychloroquine for his rheumatoid arthritis and no biologic agents were used to our knowledge. He was found to have an extra-axial mass overlying the posterior right frontal lobe at the convexity, measuring approximately 2.3 x 2.7 x 2.3 cm (anteroposterior (AP) x transverse (TR) x craniocaudal (CC)), and an intra-parenchymal lesion within the subcortical white matter of the right frontal lobe spanning approximately 0.8 x 1.1 cm with radiologic features consistent with a low-grade glial tumor (Figure 1).

**Figure 2:**
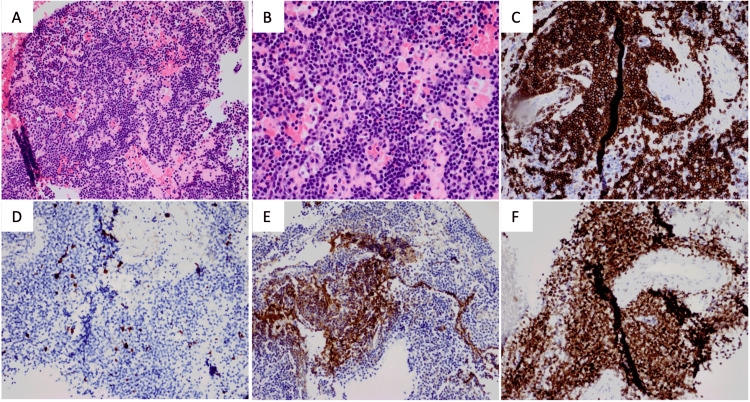
Microscopic and selected immunohistochemical stains Panels A and B show hematoxylin and eosin stain at medium (4x objective) and high power (20x), respectively. Immunohistochemistry for CD20 (C), ki67 (D), GFAP (E), and BCL2 (F) are also shown.

He was assessed by the hematology team and was cleared for surgery to resect these lesions. Neuropathologic assessment following surgical resection confirmed the diagnosis of meningioma for the convexity tumor. Histopathologic evaluation of the intraparenchymal lesion demonstrated diffuse lymphoplasmacytic infiltrate composed of small mature lymphocytes, plasmacytoid lymphocytes, and mature plasma cells. Immunohistochemistry showed that most cells were CD20-positive (B cells) with kappa light chain restriction. Ki-67 demonstrated a very modest proliferative index. The tumor was uniformly positive for BCL2 and negative for CD5, CD21, CD10, and cyclin-D1 (Figure 2). Finally, immunohistochemistry for synaptophysin and GFAP showed that the cellular infiltrate was non-reactive. This was diagnosed as an MZBCL of MALT type.

With the diagnosis of low-grade B-cell lymphoma, the patient subsequently had a bone marrow biopsy, CSF analysis, and lymph node biopsy, which did not show systemic disease. He was also found to be HIV and *H. pylori-negative*. To date, the patient has elected not to proceed with adjuvant therapies, and the nine-month follow-up MR and positron emission tomography imaging has confirmed no recurrent mass or adverse interval change within the parenchyma.

## Discussion

Only a few cases of intracranial primary low-grade lymphomas of the MALT subtype have been reported in the literature, with the vast majority located in the dura mater. As the CNS does not contain any mucosal or MALT tissue, it has been hypothesized that the meningothelial cells in the brain are analogous to epithelial cells at other sites where MALT lymphoma typically arises [8]. However, it remains unclear how primary MZBCL can manifest in intraparenchymal tissue. Recently, an association has been found between autoimmune disease and MZBL [9]. Our patient had a history of both rheumatoid arthritis and antiphospholipid syndrome, giving rise to the possibility of a causative antigen stimulus process.

A review of the literature using the EMBASE and MEDLINE databases using the keywords of “MALT lymphoma” or “mucosa-associated lymphoid tissue lymphoma” and “brain tumors” primarily yielded previous cases of dural MALT lymphomas. In total, 13 cases were found in the literature of cases who were diagnosed with primary MZBCL of MALT type involving the brain parenchyma (Table 1) [7,8,10-16]. Five cases involved the frontal cortex, four in the parietal cortex, two involved the basal ganglia, and one the midbrain. The remaining studies reported a patient with multiple lesions involving the temporal and occipital cortex, as well as the spinal cord. With the exception of the patient diagnosed post-mortem via autopsy, all cases utilized either radiation or chemotherapy with only one patient receiving both radiation and surgery.

**Table 1:** Clinical summary of patients all with a final diagnosis of intraparenchymal MZBCL from the literature M: male; F: female; NED: no evidence of disease; AWD: alive with disease; DWD: dead with disease; NR: not reported; N/A: not applicable; MZBCL: marginal zone B-cell lymphoma

Age/Gender	Location	Tumor Size	Presentation	Treatment	Outcome
66/M [7]	Right frontal cortex	NR	Seizures	Surgery and Radiation	NED at 13 months
48/M [8]	Left frontal cortex	2.0 x 1.4 x 1.6 cm	Seizures and bilateral upper extremity weakness	Radiation	NED at 15 months
18/M [10]	Left basal ganglia	4.0 x 3.5 cm	Right-sided central facial nerve palsy, weakness, dizziness, and dysarthria	Radiation	NED at 22 months
70/M [11]	Left posterior putamen, posterior subinsular region, mid-temporal region	3.5 x 2.9 cm	Dysnomia, right arm and leg numbness, slurred speech, and blurry vision	Chemotherapy	No change in residual mass at 24 months
45/M [12]	Right parietal cortex	NR	Seizures, left-sided weakness, and slurred speech	Radiation	NED at 64 months
39/F [13]	Frontal lobe cortex	NR	Vision defects with nystagmus	Chemotherapy	AWD at 24 months
53/M [14]	Right temporal, left occipital lobe, spinal cord	NR	Memory and gait disturbance with urinary incontinence	Chemotherapy	NED, unknown time
65/F [15]	Parietal lobe	NR	Diagnosed post-mortem via autopsy	N/A	N/A
NR/M [15]	Parietal lobe	NR	NR	Chemotherapy	DWD at 38 months
NR/F [15]	Parietal lobe	NR	NR	Chemotherapy	AWD at 15 months
NR/F [15]	Frontal lobe	NR	NR	Radiation	DWD at 61 months
NR/F [15]	Frontal lobe	NR	NR	Chemotherapy	AWD at 40 months
33/M [16]	Left midbrain	1.9 x 1.8 cm	Left ptosis, right limb numbness, and weakness	Radiation	NED at 6 months
64/M Present Case	Right frontal cortex	0.8 x 1.1 cm	Left-hand weakness and focal motor seizure in arm	Surgery	NED at 9 months

In addition to presenting a novel and interesting radiologic diagnosis, this case poses questions regarding the oncologic management of intraparenchymal MALT lymphomas. Currently, surgery, chemotherapy, radiation, or a combination of these modalities are used to treat CNS lymphomas. To our knowledge, our case represents the first reported instance of a patient with intraparenchymal MZBCL of MALT type to be managed solely with surgical resection. Our patient has remained disease-free at nine months postoperatively suggesting that adjuvant treatment may not be required in the initial management of intracranial MALT lymphoma in certain cases. Radiation and chemotherapy may cause significant neurotoxicity. Hence, cases need to be carefully evaluated on an individual basis to minimize iatrogenic sequelae from exposure to these therapies. Furthermore, given the propensity of MALT lymphomas to recur, this patient and others similarly affected, ought to be closely followed with serial MR imaging [17].

## Conclusions

In conclusion, the results presented here indicate that primary MZBCL may not be isolated to the meninges and can develop in brain parenchyma. Thus, MZBCL should be considered in the differential diagnosis of intra-axial CNS masses. This case suggests that localized MZBCL may be managed with local excision without the need for early radiation or chemotherapy. However, more evidence is required to draw conclusions regarding the optimal management of this disease.
